# Accurate plant 3D reconstruction and phenotypic traits extraction via stereo imaging and multi-view point cloud alignment

**DOI:** 10.3389/fpls.2025.1642388

**Published:** 2025-09-30

**Authors:** Zhencan Wang, Huichun Zhang, Liming Bian, Lei Zhou, Yufeng Ge

**Affiliations:** ^1^ College of Mechanical and Electronic Engineering, Nanjing Forestry University, Nanjing, Jiangsu, China; ^2^ Jiangsu Co-Innovation Centre of Efficient Processing and Utilization of Forest Resources, Nanjing Forestry University, Nanjing, Jiangsu, China; ^3^ State Key Laboratory of Tree Genetics and Breeding, Co-Innovation Centre for Sustainable Forestry in Southern China, Key Laboratory of Forest Genetics & Biotechnology of Ministry of Education, Nanjing Forestry University, Nanjing, Jiangsu, China; ^4^ Department of Biological Systems Engineering, University of Nebraska-Lincoln, Lincoln, NE, United States; ^5^ Center for Plant Science Innovation, University of Nebraska-Lincoln, Lincoln, NE, United States

**Keywords:** plant phenotyping, 3D reconstruction, point cloud alignment, stereo imaging, SfM-MVS

## Abstract

**Introduction:**

Accurate 3D reconstruction is essential for plant phenotyping. However, point clouds generated directly by binocular cameras using single-shot mode often suffer from distortion, while self-occlusion among plant organs complicates complete data acquisition.

**Methods:**

To address these challenges, this study proposes and validates an integrated, two-phase plant 3D reconstruction workflow. In the first phase, we bypass the integrated depth estimation module on camera and instead apply Structure from Motion (SfM) and Multi-View Stereo (MVS) techniques to the captured high-resolution images. It produces high-fidelity, single-view point clouds, effectively avoiding distortion and drift. In the second phase, to overcome self-occlusion, we register point clouds from six viewpoints into a complete plant model. This process involves a rapid coarse alignment using a marker-based Self-Registration (SR) method, followed by fine alignment with the Iterative Closest Point (ICP) algorithm.

**Results:**

The workflow was validated on two Ilex species (Ilex verticillata and Ilex salicina). The results demonstrate the high accuracy and reliability of the workflow. Furthermore, key phenotypic parameters extracted from the models show a strong correlation with manual measurements, with coefficients of determination (R²) exceeding 0.92 for plant height and crown width, and ranging from 0.72 to 0.89 for leaf parameters.

**Discussion:**

These findings validate our workflow as an accurate, reliable, and accessible tool for quantitative 3D plant phenotyping.

## 1 Introduction

Plant phenotyping refers to the determination of quantitative or qualitative values for morphological, physiological, biochemical, and performance-related properties, which act as observable proxies between gene(s) expression and environment and are important determinants of growth, quality, and stress resistance characteristics (Bian et al., 2022). Traditional phenotyping relies on visual observation and manual measurements, which is labor-intensive and highly experience-dependent. In recent years, imaging technology has become an effective tool for studying plant phenotypes, including visible-light, spectral (both multispectral and hyperspectral), thermal, and fluorescence cameras (Zhang et al., 2023).

Among the various phenotypic traits, morphological and structural characteristics directly reflect plant growth (Song et al., 2023), which is why most current phenotypic studies focus on assessing these traits. Traditional 2D image-based analysis methods project the 3D spatial structure of the plant onto a 2D plane, which results in the loss of depth information and fails to accurately capture the plant’s morphological features. One noteworthy development is the adoption of 3D plant phenotyping methods (Vázquez-Arellano et al., 2016). In some cases, 3D sensing methods that incorporate data from multiple viewing angles may provide information and insights that are hard or impossible to get from a 2D model alone (Harandi et al., 2023). Current 3D imaging techniques applied in phenotyping mainly include image-based method, laser scanning-based method, and depth camera-based method (Song et al., 2023).

Light detection and ranging (LiDAR) as a sophisticated active remote sensing technology, acquires high-precision three-dimensional point cloud data by emitting laser pulses and measuring their return times with great accuracy (Lin, 2015; Jin et al., 2021). This capability offers notable advantages in plant phenotyping studies. For instance, research on cotton has demonstrated that ground-based LiDAR can measure traits such as main stem length and node count with accuracy comparable to manual methods (Sun et al., 2021). However, two key challenges still hinder its broader application: (1) capturing the complete 3D structure of plants often requires multi-site scanning and subsequent fusion of multi-view point cloud data; (Li et al.,2023; Liu et al., 2025) and (2) the high cost of LiDAR equipment remains a significant barrier to its widespread adoption.

Image-based reconstruction techniques primarily use the structure from motion (SfM) algorithm, which reconstructs a 3D point cloud by matching feature points across multiple 2D images (Song et al., 2023). Wang et al. (2022) used 100 images around tomato plants for 3D point cloud reconstruction; while Li et al. (2022) used 50–60 images for the 3D reconstruction of maize plants. The number of required images depends on plant height and phenotyping needs—smaller plants may require about 60 images, while taller ones may need up to 80 (Hui et al., 2018). Although image-based methods can produce detailed point clouds with low-cost equipment, they are time-consuming and computationally intensive, limiting their application in high-throughput phenotyping.

Depth camera-based techniques offer an alternative for acquiring point clouds. Unlike image-based methods, depth cameras directly capture depth images (point clouds) without the need for metric conversion (Yang et al., 2022). Depth cameras are typically classified into two categories based on their operating principles: time of flight (ToF)-based and binocular stereo vision-based. ToF cameras use light emitted by a laser or LED source and measure the roundtrip time between the emission of a light pulse and the reflection from thousands of points to build up a 3D image (Kolhar and Jagtap, 2023). ToF cameras are widely used in morphological phenotyping to measure plant height (Jiang et al., 2016; Ma et al., 2019), leaf area (Chéné et al., 2012; Song et al., 2023), etc. However, their relatively low resolution can miss fine details, especially for smaller plants or delicate structures like stalks and petioles (Paulus, 2019).

Binocular stereo vision cameras (stereo cameras) use two or more lenses and separate image sensors to capture two slightly different images, allowing 3D structure reconstruction by calculating the distance from pixel disparities (Li et al., 2016). However, Due to the inherent limitations of binocular camera hardware and the texture-based matching principles of their imaging process, feature extraction on low-texture or smooth surfaces (such as calibration spheres) is significantly constrained. This often leads to point cloud distortions; for example, a reconstructed calibration sphere may appear as a flat circle rather than a three-dimensional hemisphere. Moreover, inherent boundary effects in disparity calculations along the edges of complex, non-rigid objects—such as plant leaves—combined with their curved surface geometry and frequent local occlusions, further exacerbate feature matching errors. These issues typically manifest as point cloud drift, such as layered noise along leaf edges. Collectively, these factors limit the accuracy of 3D reconstruction.

Due to mutual occlusions between plant organs, obtaining a complete 3D point cloud of the plant from a single viewpoint scan is challenging, regardless of whether LiDAR or other 3D imaging technologies. To address this, a registration algorithm is essential to align point clouds from different coordinate systems into a single system, eliminating occlusion and ensuring a complete point cloud (Teng et al., 2021). Fusing point clouds obtained from multiple angles is a common method to establish accurate 3D models, and researchers tend to reconstruct plant models through point clouds data from three or more angles (Hu et al., 2018; Moreno et al., 2020). The premise of point cloud fusion is to realize the registration of multiple point clouds, which accurately align the point cloud data from different views into a complete 3D model of the plant (Li and Tang, 2017). For example, Yang et al. (2022) introduced a self-registration method for tree seedlings using calibration objects on a precision turntable, demonstrating that high-precision 3D models could be achieved with low-cost equipment. Likewise, Li et al. (Xiuhua et al., 2021). and Chen et al. (Haibo et al., 2022) utilized multi-view point cloud fusion and registration techniques to reconstruct banana seedlings and other plant models effectively. Despite the advancements in multi-view point cloud fusion, these reconstructions still lag behind image-based methods in terms of accuracy. Therefore, most studies focus on morphological phenotyping at the plant scale, such as plant height (Xiuhua et al., 2021; Haibo et al., 2022; Yang et al., 2022), and crown width (Li and Tang, 2017), with fine-scale phenotypic traits, such as leaf length and leaf width, rarely being addressed.

This paper proposes and validates an automated workflow for Binocular stereo vision-based three-dimensional fine-grained reconstruction and phenotyping of plants. The core contributions are as follows: (1) high-resolution RGB images captured by a stereo camera are combined with SfM and MVS algorithms to reconstruct single-view point clouds of the plant; (2) a self-registration (SR) algorithm based on a calibration sphere is employed for the initial alignment of multi-view point clouds, followed by fine registration using the Iterative Closest Point (ICP) algorithm, resulting in a unified and complete 3D plant model. Based on this model, four key phenotypic parameters—plant height, crown width, leaf length, and leaf width—are automatically extracted. Comparative analysis with manually measured data demonstrates the high accuracy and reliability of the proposed method.

## 2 Materials and methods

### 2.1 Image acquisition system for seedlings

**Figure 1:**
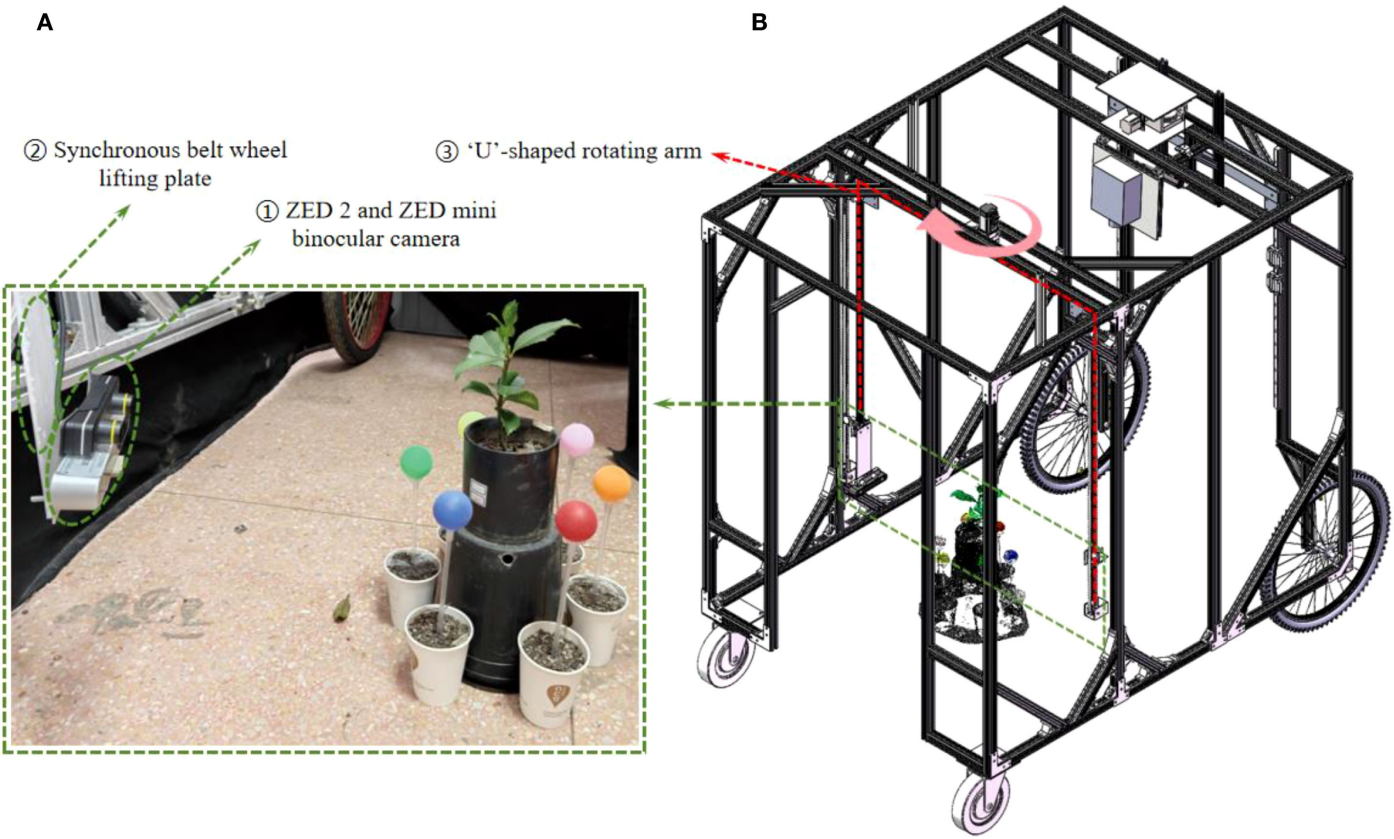
3D reconstruction system for tree seedlings. **(A)** A working diagram of the 3D reconstruction system on site. **(B)** Solidworks drawing of 3D reconstruction system. The red dotted box indicates the U-shaped rotating arm module, and the pink curved arrow shows the rotation direction of the platform. The green dotted box highlights the camera acquisition module, which corresponds to the field acquisition diagram of the camera module. The oval green line box indicates the ZED camera and the timing belt lifting mechanism, respectively.

**Figure 2:**
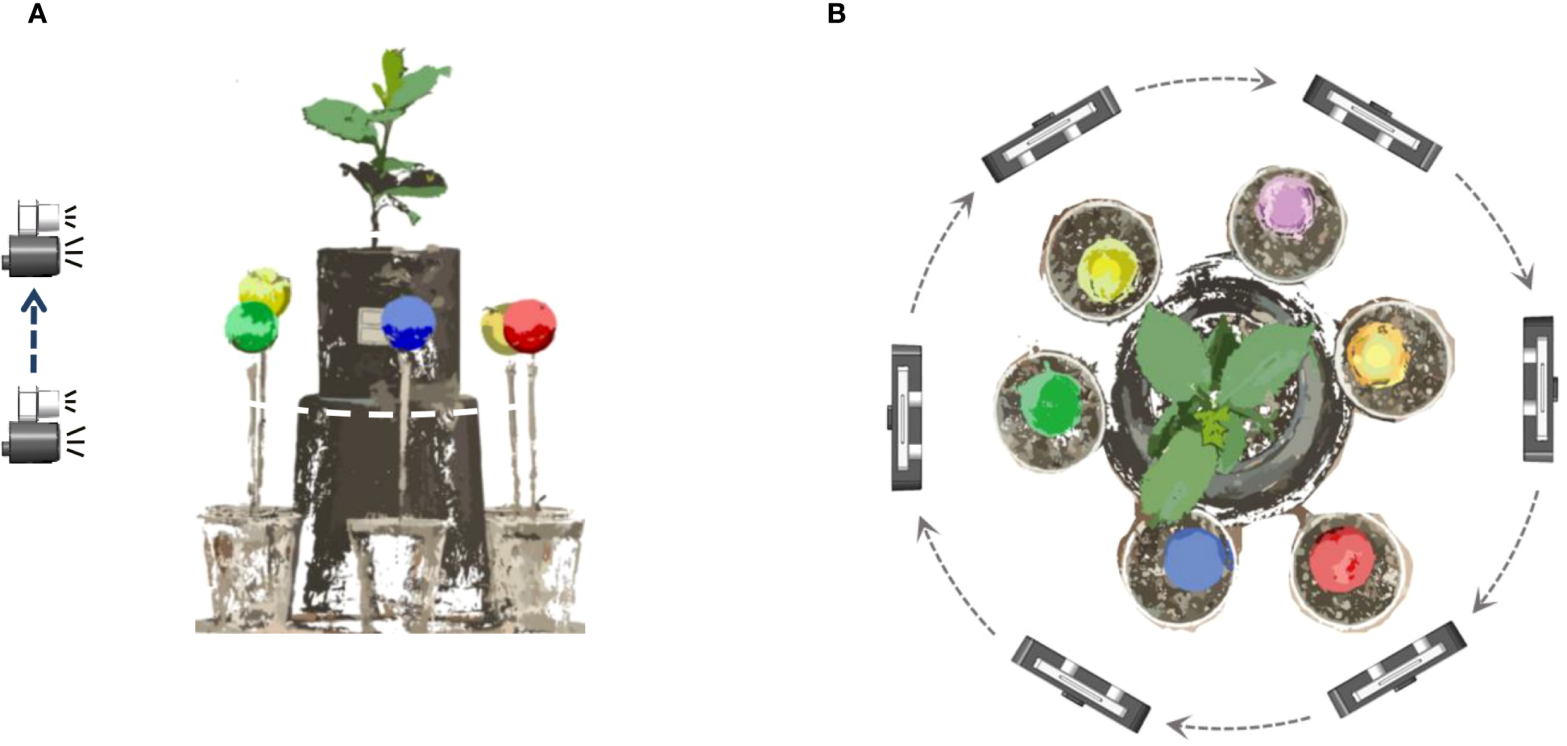
Diagram of data acquisition method. **(A)** The image acquisition process from a single viewpoint. **(B)** Image acquisition process at 6 viewing angles.

**Figure 1** illustrates the self-developed seedling reconstruction system, which mainly consists of a ‘U’-shaped rotating arm, a synchronous belt wheel lifting plate, a ZED 2 and a ZED mini binocular camera as the image acquisition device (marked as ① in **Figure 1**). The ZED mini is mounted on the ZED 2, which can simultaneously capture 4 images with a resolution of 2208×1242 in a single shot. The synchronous belt wheel lifting plate enables vertical movement of the camera system, allowing image capture from various heights. In this study, images were acquired twice at the same viewing angle, resulting in a total of 8 RGB images. The image acquisition process from a single viewpoint is illustrated in **Figure 2A**.

The ‘U’-shaped rotating arm allows for 360° rotation of the platform around the plant. After acquiring images from one viewpoint, the arm rotates by 60° and pauses before the next round of image capture, as shown in **Figure 2B**. This rotation strategy enables image capture from six distinct angles: 0° (360°), 60°, 120°, 180°, 240°, and 300°, yielding a total of 48 images. The acquired images were then transmitted to the image workstation via a Jetson Nano edge computing device (NVIDIA), which is powered by an AMD Ryzen 9 5900X 12-core processor with 32GB of RAM running Windows 10, alongside an NVIDIA GeForce RTX 3080Ti GPU with 12GB of video memory.

To effectively perform multi-view point cloud registration, six passive spherical markers (calibration spheres, commercially available) with a known diameter and matte, non-reflective surfaces are positioned at equal distances around the plant, as shown in **Figure 1A**. The different colors of the calibrators facilitate their segmentation and subsequent point cloud alignment.

### 2.2 Single-view point cloud reconstruction via SFM-MVS

**Figure 3:**
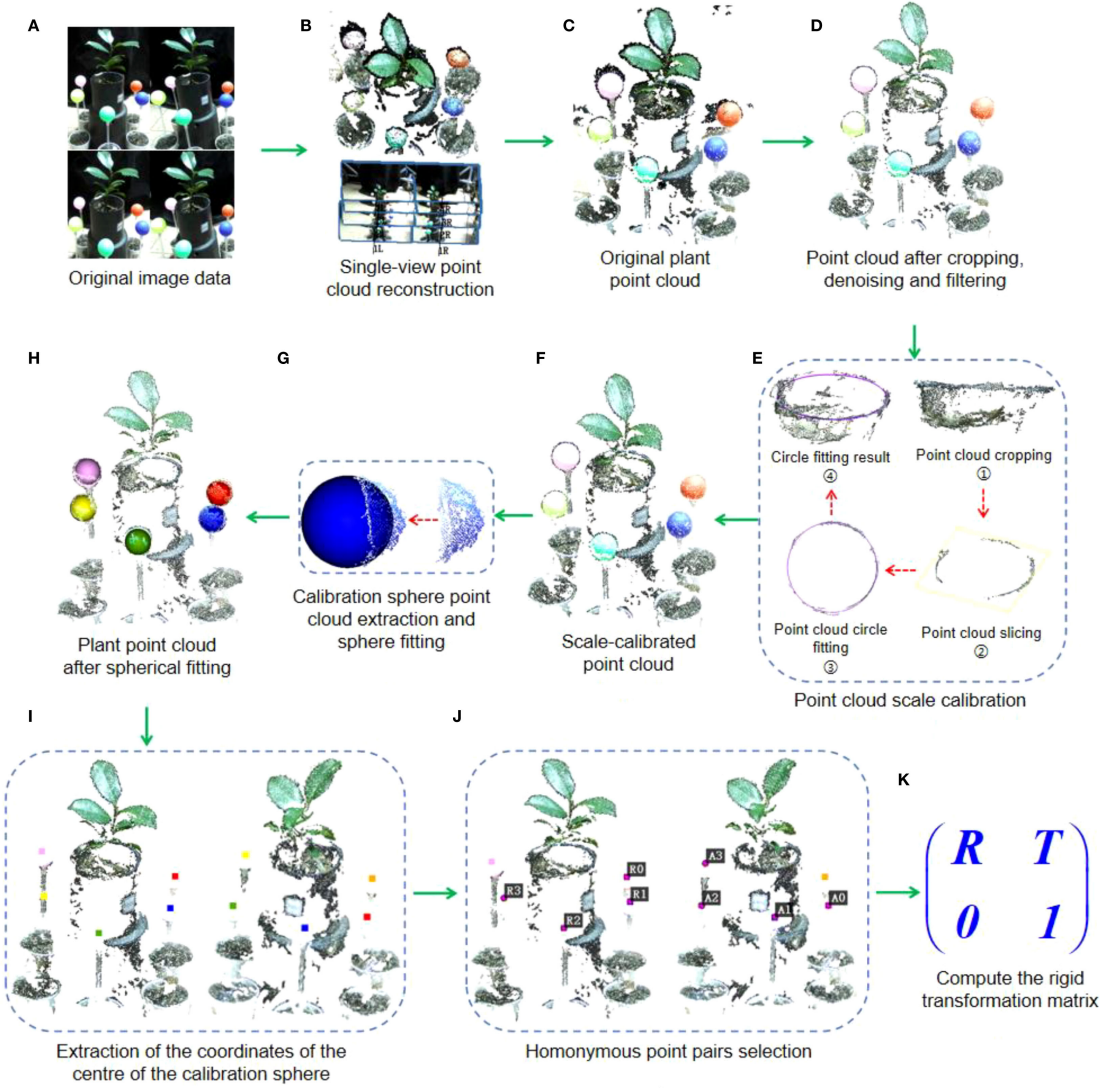
Calculation process of rigid transformation matrix for adjacent point clouds. **(A)** Original image data acquired by the binocular camera. **(B)** Single view point cloud reconstruction process. **(C)** Reconstructed original point cloud. **(D)** Point cloud after cropping, denoising and filtering. **(E)** Point cloud scale correction process. **(F)** Scale-calibrated point cloud. **(G)** Calibration sphere point cloud extraction and sphere fitting. **(H)** Plant point cloud after spherical fitting. **(I)** Extraction of the coordinates of the centre of the calibration sphere. **(J)** Homonymous pointpairs selection. **(K)** Compute the rigid transformation matrix.

Binocular stereo cameras are often used to directly obtain point cloud from a single perspective of plants, e.g., sorghum (Bao et al., 2019; Xiang et al., 2021), maize (Xiang et al., 2023), etc. However, due to limitations inherent in the imaging hardware and the depth map generation mechanism, plant point clouds captured directly using the official SDK often exhibit shape distortions and spatial drift. To overcome these issues, our workflow processes the images from each of the six viewpoints independently. For each viewpoint, the set of 8 captured RGB images was input into Agisoft Metashape to execute a complete SfM-MVS reconstruction, yielding a high-fidelity single-view point cloud (as illustrated in **Figures 3A–C**). This process was repeated for all six viewpoints, resulting in six separate point clouds ready for registration. In contrast to the official SDK, which performs real-time depth estimation based on a single image pair, our offline approach incorporates global optimization and cross-view consistency checks. This method yields a point cloud with substantially improved geometric accuracy and reduced noise, offering a more robust and reliable foundation for downstream phenotyping analysis.

### 2.3 Point cloud pre-processing

The reconstructed point clouds typically contain significant noise, particularly black noise in the edge areas of plant leaves. This noise is visually distinct from the plant and calibration sphere, making it possible to filter out using a color-based filtering algorithm. In this paper, we employ the R (Red), G (Green), and B (Blue) color space, setting threshold values for each channel at 50 to filter out black or dark gray noise. Following this, further noise, including outliers, is removed by cropping and denoising, resulting in a cleaner point cloud, as shown in **Figure 3D**.

### 2.4 Scale calibration for single-view point clouds

The reconstruction process described in Section 2.3 yields six high-fidelity point clouds, each representing a different viewpoint of the plant. However, a fundamental characteristic of the SfM pipeline is that each reconstruction using SFM-MVS algorithms or software are scaled (Rose et al., 2015), therefore it is essential to standardize the scale of point clouds from various perspectives before alignment.

To maximize geometric accuracy, we employed an offline SfM-MVS pipeline instead of the camera’s real-time depth estimation. Since this method produces point clouds without a metric scale, we established the correct scale for each view using the known diameter of the plant pot. As shown in **Figure 3E**, the top of pot is cropped, and a set of circular ring point clouds is derived using point cloud slicing algorithm. Then, the radius of the pot is determined by fitting a circle to the segmented point cloud. Assuming that the estimated pot diameters in the six viewpoints are γ0, γ1, γ2, γ3, γ4, and γ5, respectively, with γ2 as the reference, we calculate the scaling ratios for each fitted circle relative to γ2 as: μ0=γ0/γ2, μ1=γ1/γ2, μ3=γ3/γ2, μ4=γ4/γ2, μ5=γ5/γ2. Then the scale factors μ0 ~μ5 are applied to the corresponding plant point cloud for scaling, thus completing the scale calibration of the multi-view point cloud.

### 2.5 Calculation of calibration sphere centre coordinates

The precise estimation of the spherical center coordinates plays a crucial role in determining the point cloud’s positional attitude and computing the transformation matrix. To achieve this, we employ an improved Random Sample Consensus (RANSAC) ball fitting algorithm to evaluate the sphere center coordinates of the calibrated ball.

The original RANSAC algorithm is vulnerable to the influence of outlier points during ball center estimation, which results in decreased accuracy. To mitigate the adverse effects of outliers, we optimize the original RANSAC algorithm by introducing dynamic interior thresholding, multi-stage optimization, and geometric constraints. The dynamic interior point threshold mechanism adjusts the criteria for determining interior points according to the actual data distribution, thus avoiding errors caused by fixed thresholds that cannot accommodate complex data patterns. Multi-stage optimization enhances the accuracy of sphere coordinate calculation by progressively refining the fitting results. Geometric constraints integrate the sphere’s geometric properties to further limit the possible solution space, thereby improving the accuracy of the sphere center calculation.

The specific calculation flow of this enhanced algorithm is illustrated as follows (**Algorithm 1**). This provides a more reliable approach for the precise estimation of sphere center coordinates.

Algorithm 1 **Enhanced RANSAC algorithm.**


Input: Calibration sphere point cloud *P*, maximum number of iterations *N*, baseline threshold *t_base_
*.
Output: Sphere center coordinates *C* (*x, y, z*) and radius *r*.
1.Initialize best model parameters: *C_best_
* = None, *r_best_
*= 0, score*
_best_
*= 0
2.For *i* = 1 to *N*:
3. Randomly sample 4 non-coplanar points to generate candidate sphere *C_candidate_
*, *r_candidate_
*
4. Compute local density, dynamically adjust threshold *t* = *f* (*t_base_
*, density)
5.Count inliers: inliers = 
{p∈P‖distance(p,Ccandidate)-rcandidate|≤t}

6. Compute the average distance μ and standard deviation σ of the inliers
7. Compute model weight: 
$w=\frac{\text{len}(\text{inliers})}{\text{len}(P)}/(\mu +\sigma )$
8. if *w>* score*
_best_
*, update *C_best_
*, *r_best_
*, score*
_best_
*
9.Apply geometric constraints (such as sphere center range filtering) to the top-K candidate models
10.Use Levenberg-Marquardt (LM) algorithm to optimize *C_best_
*, *r_best_
* for the final model’s inliers
11.Return *C_best_
*, *r_best_
*

### 2.6 Calculation of the transformation matrix by means of the SR

**Figure 4:**
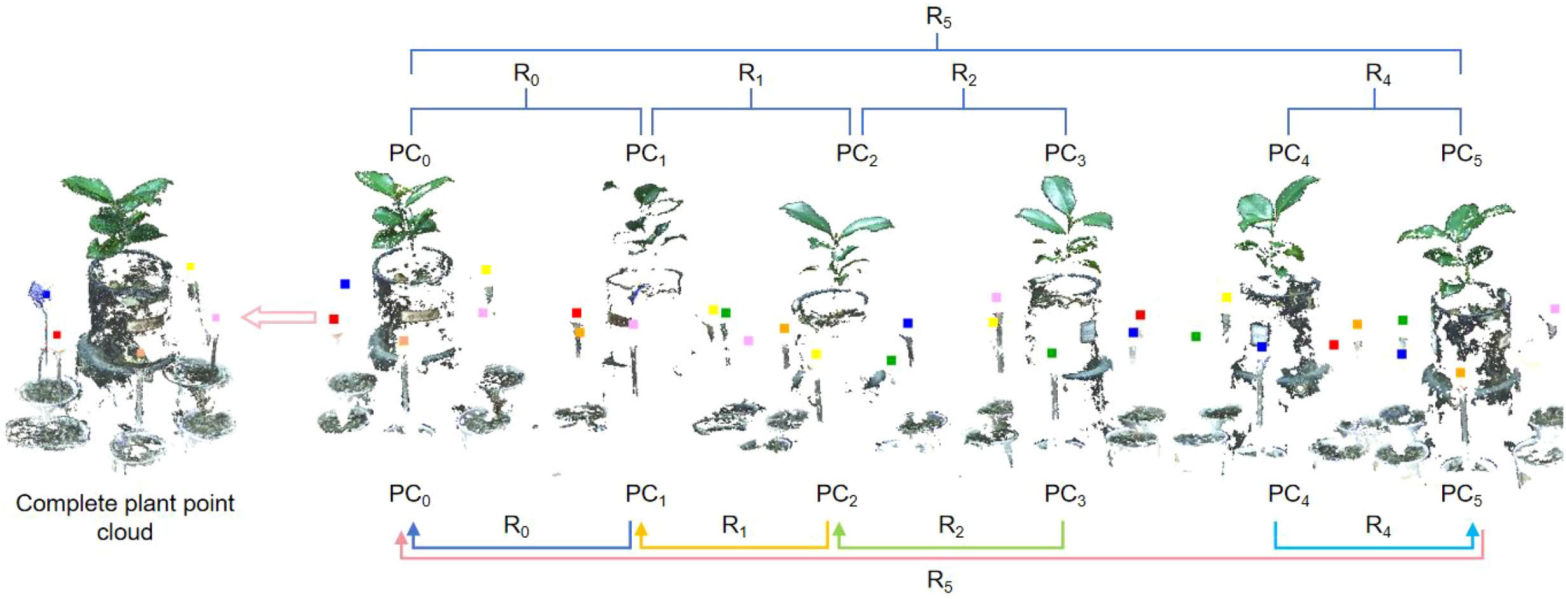
Compute the transformation matrix between two homonymous point sets. **(A)** Point clouds generated directly by the binocular camera’s official SDK. **(B)** Point clouds generated by off-line SfM-MVS pipeline.

1. Determine the set of homonymous points. For the two adjacent point clouds, at least three calibration sphere centers should overlap, which define the set of homonymous points. In this paper, the set of coordinates of the centre of the calibration sphere in the 0° viewing angle is denoted by PC_0_, and 60° ~ 300° are represented as PC_1_~PC_5_ respectively.
2. Transformation Matrix Calculation. To align the point clouds from different viewpoints, the transformation matrix between each homonymous point set must be calculated. Set PC_0_ as the reference point set, and PC_1_~PC_5_ as aligned point set. The transformation matrix between PC_1_ and PC_0_ can be calculated by Singular Value Decomposition (SVD). By the same token, the transformation matrices between the corresponding point set PC_2_ and PC_1_, PC_5_ and PC_4_, PC_3_ and PC_2_ can also be acquired. The transformation matrix between the corresponding homonymous point sets is denoted by R_0_, R_1_, R_2_, R_4_, and R_5_, is illustrated in **Figure 4**.

**Figure 5:**
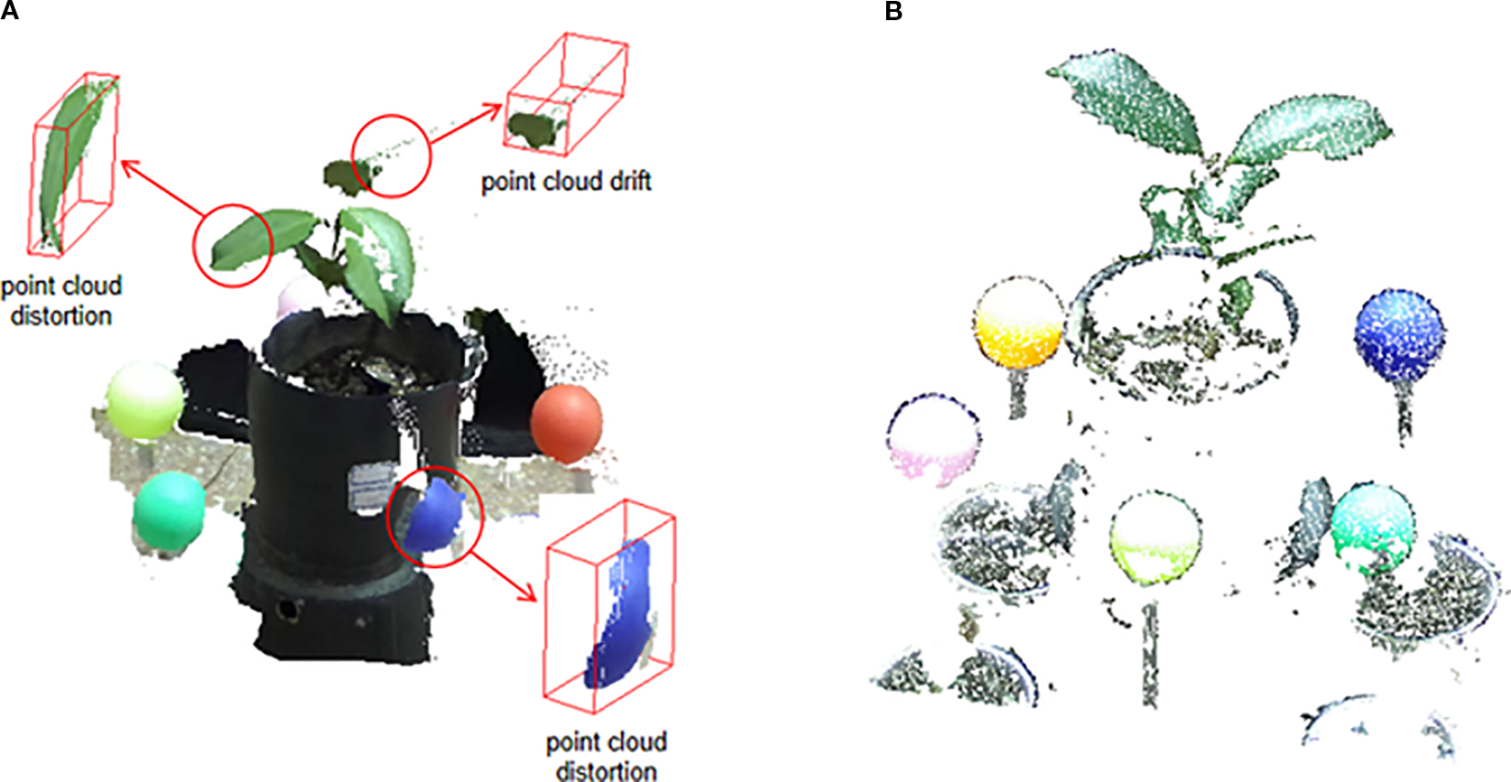
Comparison of point cloud reconstruction results. **(A)** Point clouds generated directly by the binocular camera’s official SDK. **(B)** Point clouds generated by off-line SfM-MVS pipeline.

## 3 Results and analysis

### 3.1 Comparison and analysis of point cloud reconstruction results

As discussed, point clouds generated directly by the binocular camera’s official SDK often suffer from significant quality issues. This is starkly illustrated in **Figure 5A**. The point cloud, derived from the camera’s real-time stereo matching and triangulation, exhibits severe geometric distortion. For example, the spherical calibrator is incorrectly reconstructed as a flattened, disk-like shape, and the plant leaves are plagued by layered noise and spatial drift at their edges. These artifacts are characteristic limitations of real-time, two-view stereo algorithms when dealing with texture-poor surfaces and complex object boundaries.

In sharp contrast, our proposed workflow, which processes the same raw RGB images using an offline SfM-MVS pipeline, yields substantially superior results, as shown in **Figure 5B**. The reconstructed point cloud is geometrically accurate and clean. The plant contours are well-defined with minimal artifacts, and crucially, the calibration sphere is reconstructed with its correct three-dimensional hemispherical shape, showing minimal distortion. This qualitative comparison highlights a key contribution of our work: by replacing the camera’s native, real-time depth estimation with a more robust, globally optimized SfM-MVS approach, we effectively mitigate the issues of distortion and drift, producing a high-fidelity point cloud that serves as a reliable basis for subsequent registration and analysis.

### 3.2 Point cloud preprocessing results

As described in Section 2.3, the point cloud preprocessing results are shown in **Figure 3D**. The preprocessing steps, including noise removal and filtering. The preprocessing improves the overall quality of the point cloud, ensuring that it is ready for further processing steps such as calibration and registration.

### 3.3 Point cloud calibration

**Figure 6:**
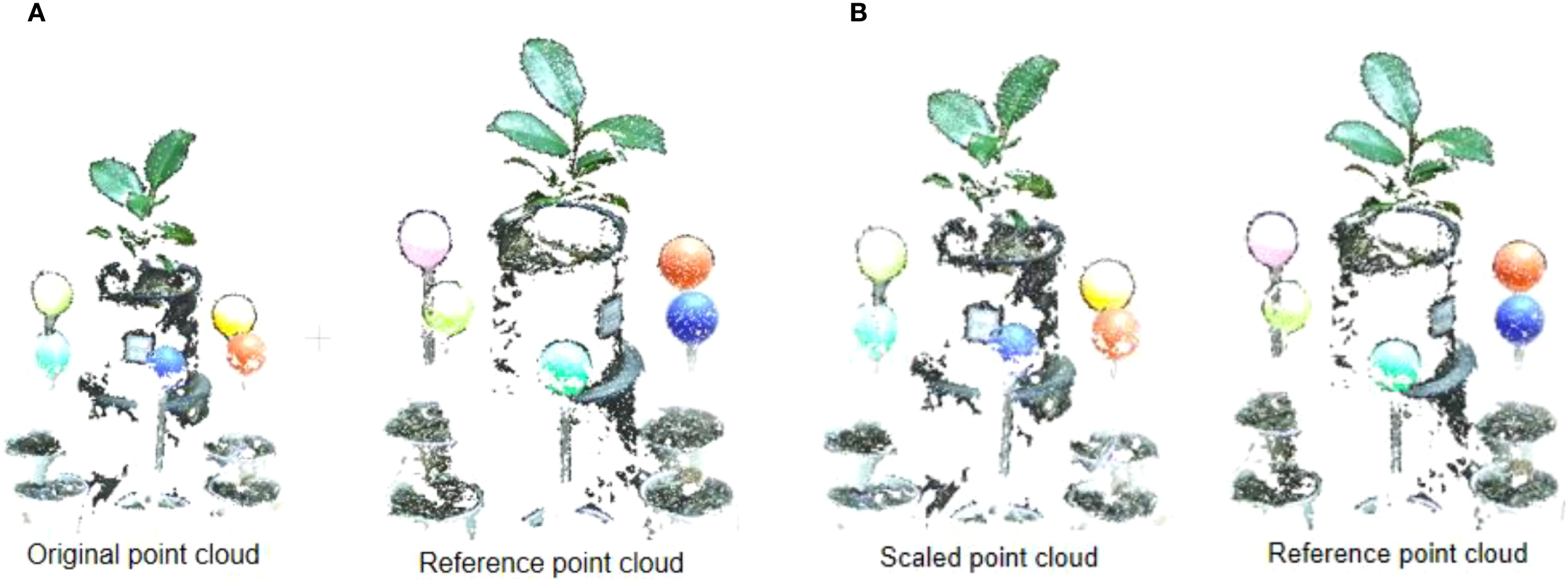
The process point cloud calibration. **(A)** Original point cloud and reference point cloud before point cloud calibration. **(B)** Original point cloud and reference point cloud after point cloud calibration.

Before point cloud calibration, the plant point clouds obtained from different perspectives were inconsistent in scale. After calibration, the sizes of point clouds in each group are consistent, ensuring a uniform scale. **Figure 6** shows the point cloud before and after calibration in two adjacent viewpoints. The reference point cloud is the point cloud used as a reference and its scale is set to 1, and the scaled point cloud is the one that is scaled relative to the reference point cloud.

### 3.4 Calculation of calibration sphere center

**Figure 7:**
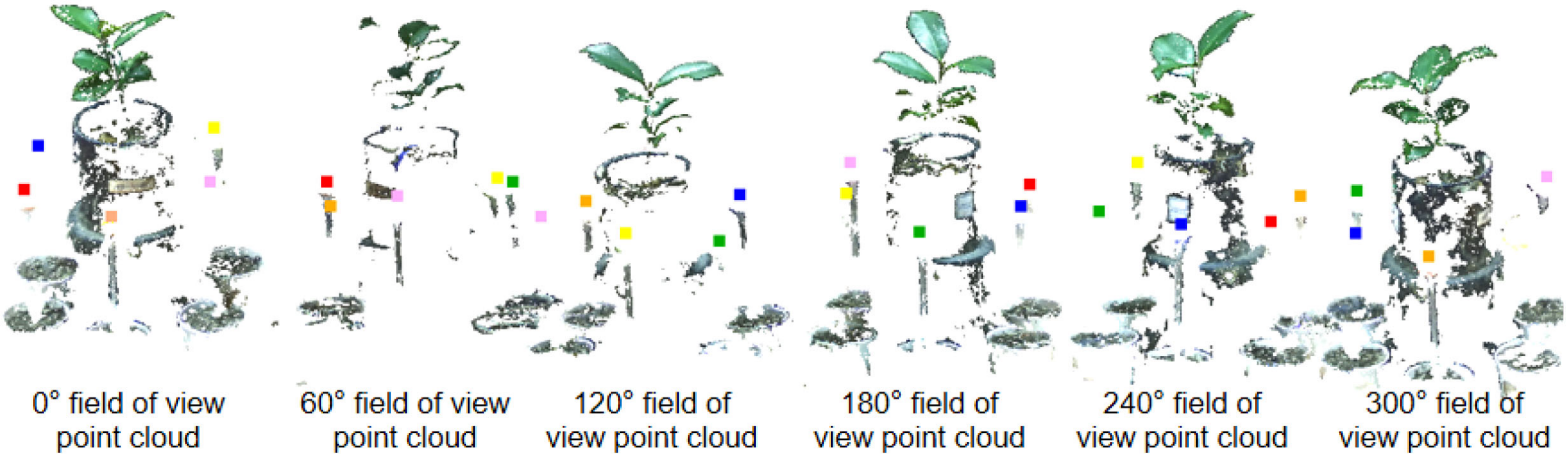
Calculation results for the center of the calibration sphere in multi-view point cloud.

The center of the calibration sphere was determined using the enhanced RANSAC algorithm, as described in Section 2.5. Through iterative looping, the coordinates of the calibration sphere’s center were calculated, as shown in **Figure 7**.

### 3.5 Point cloud registration results

**Figure 8:**
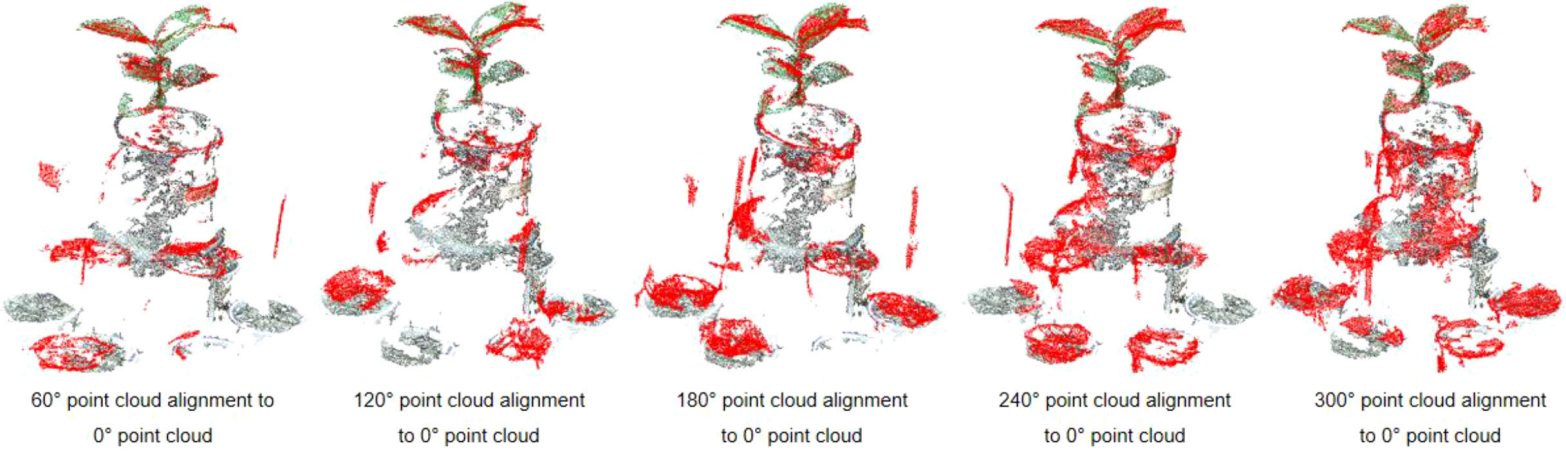
The SR-ICP registration method described in this article.

The plant point cloud collected from a 0° perspective is taken as the reference point cloud. The point clouds from other perspectives are transformed to the spatial coordinate system of 0° perspective through the rigid transformation matrix calculated by the calibration spheres. The specific process is as follows: Rotate and translate the point cloud from 60° perspective to the 0° point cloud coordinate system through the transformation matrix R_0_. Afterward ICP algorithm is used for fine registration, ensuring precise alignment between the point clouds from both perspectives. We set the maximum number of iterations to 100, with an RMSE (Root Mean Square Error) threshold of 1e-8. The final overlap achieved was 50%, resulting in a good configuration. Similarly, the point cloud from 120°can be transform to 0°coordinate system by transformation matrix R_0_×R_1_, followed by fine alignment with the ICP algorithm. The same process was applied to the other viewpoints, with the final alignment results displayed in **Figure 8**.

Compared to a single-perspective point cloud acquisition, the multi-view point cloud registration approach fills in the holes and gaps in the original point cloud, significantly enhancing the accuracy and completeness of the reconstructed 3D model. This method ensures that all surfaces of the plant are captured and represented in the final point cloud.

## 4 Discussion

### 4.1 The underlying mechanisms of distortion and drift elimination

The significant improvement in point cloud quality achieved by our method (as illustrated in **Figure 5**) stems from a fundamental paradigm shift: replacing the camera’s built-in, real-time, local matching algorithm with an offline, globally optimized 3D reconstruction workflow.

To ensure real-time performance, the stereo matching algorithm used by the camera SDK estimates depth from individual image pairs. However, this approach is highly prone to matching ambiguities in low-texture surfaces (e.g., calibration spheres) and complex boundaries (e.g., plant leaf edges), often resulting in geometric distortions and edge drift. Because the computation is local and pairwise, such errors are difficult to correct.

In contrast, our SfM-MVS workflow processes all images from a single viewpoint as a unified dataset. At the core of SfM is bundle adjustment, a global optimization process that enforces geometric consistency across the entire image set, thereby fundamentally correcting errors introduced by local mismatches. Subsequently, in the MVS stage, redundant information from multiple viewpoints is exploited to robustly reconstruct challenging regions, including smooth surfaces and leaf edges. The result is a point cloud that offers both high geometric fidelity and structural completeness. Thus, the shift from error-prone local real-time processing to robust global optimization is the key to achieving high-accuracy reconstruction in our proposed method.

### 4.2 Evaluation of point cloud reconstruction accuracy and efficiency

MVS three-dimensional (3D) reconstruction is considered to be the optimal solution to build a high-throughput and low-cost phenotyping platform for individual plants (Wu et al., 2022). The previous studies have shown that the phenotypes retrieved from MVS reconstruction can match the accuracy of LiDAR and reconstruct a high-quality 3D point cloud with vertex colors (Wang et al., 2021). Therefore, in this paper, we compare the proposed method with MVS-based point cloud reconstruction in terms of both accuracy and efficiency.

Image-based point cloud reconstruction typically requires a high degree of overlap between adjacent images, meaning that a series of multi-view images—usually between 25 and 30 must be collected; this continuous image acquisition is time-consuming and significantly reduces the efficiency of point cloud reconstruction. In comparison, the method proposed in this study enhances both the efficiency of image acquisition and point cloud reconstruction, allowing for faster processing and high-throughput phenotyping. The image acquisition and plant point cloud generation algorithms in this article take about 6 and 7 seconds respectively in a single perspective. Therefore, the total time for point cloud reconstruction from a single viewpoint is under 15 seconds. In the process of point cloud registration, the program automatically estimates the centers of the calibration spheres and the rotation matrix. Through program iteration, the rotation and translation matrices for six angles can be computed in approximately 5 seconds. Consequently, the total time consumption of the reconstruction method proposed is approximately 100s, with a maximum of 2 minutes. While the multi-view image reconstruction method requires the acquisition of plant images from multiple viewpoints, thus, the camera must execute a sequence of “hold-capture-rotate” operations during image collection. Furthermore, the camera must be stabilized to avoid image blurring caused by camera vibration, introducing a designated dwell time before capturing each image. After testing, the collection of 25 multi-view images can yield a relatively complete plant point cloud, taking approximately 2 minutes and 30 seconds, which is about 30 seconds longer than the method proposed in this paper. In conclusion, the method proposed here can enhance the efficiency of point cloud reconstruction by over 25%.

We evaluated geometric accuracy by computing the cloud-to-cloud distance against a reference model. To isolate the analysis to the plant’s structure, non-plant objects (e.g., the pot and spheres) were removed from both clouds before comparison.

**Figure 9:**
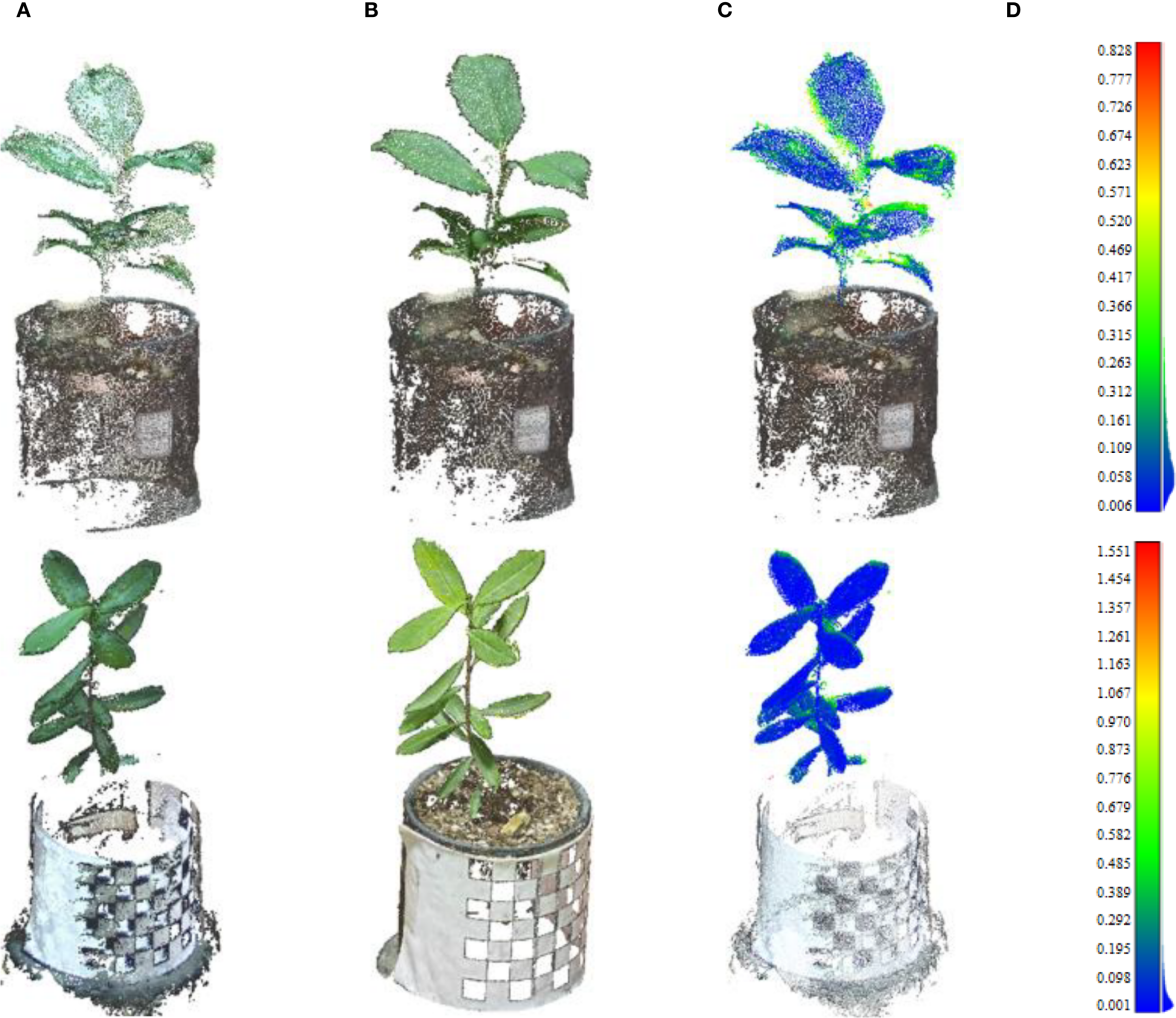
Comparative analysis of point cloud reconstruction accuracy. The point clouds reconstructed in the first row are of the Ilex verticillata variety, and those in the second row are of the Ilex salicina variety. **(A)** The complete plant point cloud was obtained after registration using the SR-ICP algorithm proposed in this study. **(b)** Point cloud obtained via MVS. **(C)** Distance heatmap between point clouds obtained via SR-ICP algorithm and MVS algorithm **(D)** Point cloud distance heatmap colbar.

In terms of reconstruction accuracy, the proposed SR-ICP workflow demonstrates a performance on par with established image-based reconstruction techniques. As illustrated in **Figure 9**, the multi-view registration strategy effectively compensates for point cloud incompleteness caused by self-occlusion in single-view acquisitions. While this superposition introduces minor misalignments at complex boundaries like leaf edges—which constitute the primary source of error—the overall geometric fidelity remains high.

A quantitative analysis of the plant-only portions of the point clouds confirms this high accuracy. For the Ilex verticillata sample, the mean and standard deviation of the distance error were 0.07 cm and 0.11 cm, respectively. For the Ilex salicina, the mean and standard deviation were 0.12 cm and 0.10 cm. These low error values demonstrate that our method achieves strong consistency with the image-based approach, validating its capacity for producing accurate and comparable 3D plant models.

A key limitation and trade-off in our proposed workflow must be acknowledged. While the image acquisition and reconstruction process are efficient, the multi-view registration step depends on the manual placement of physical calibration spheres. This marker-based registration introduces an additional setup step during data collection, which is not required in fully automated, marker-less MVS pipelines. This reflects a trade-off between automation and registration accuracy. For objects with complex geometry and severe occlusion—such as plants—marker-less registration methods often struggle, leading to alignment failures or significant cumulative drift in the absence of ground control points. We opted for the marker-based approach because it offers robust and highly accurate initial alignment, which is essential for the success of the subsequent ICP-based fine registration and the high-precision phenotyping demonstrated in this study. Future research could explore semi-automated or robust marker-less registration techniques tailored to plant structures to improve overall throughput without compromising accuracy.

### 4.3 Assessment of plant 3D phenotypic parameters based on multi-view point cloud alignment

**Figure 10:**
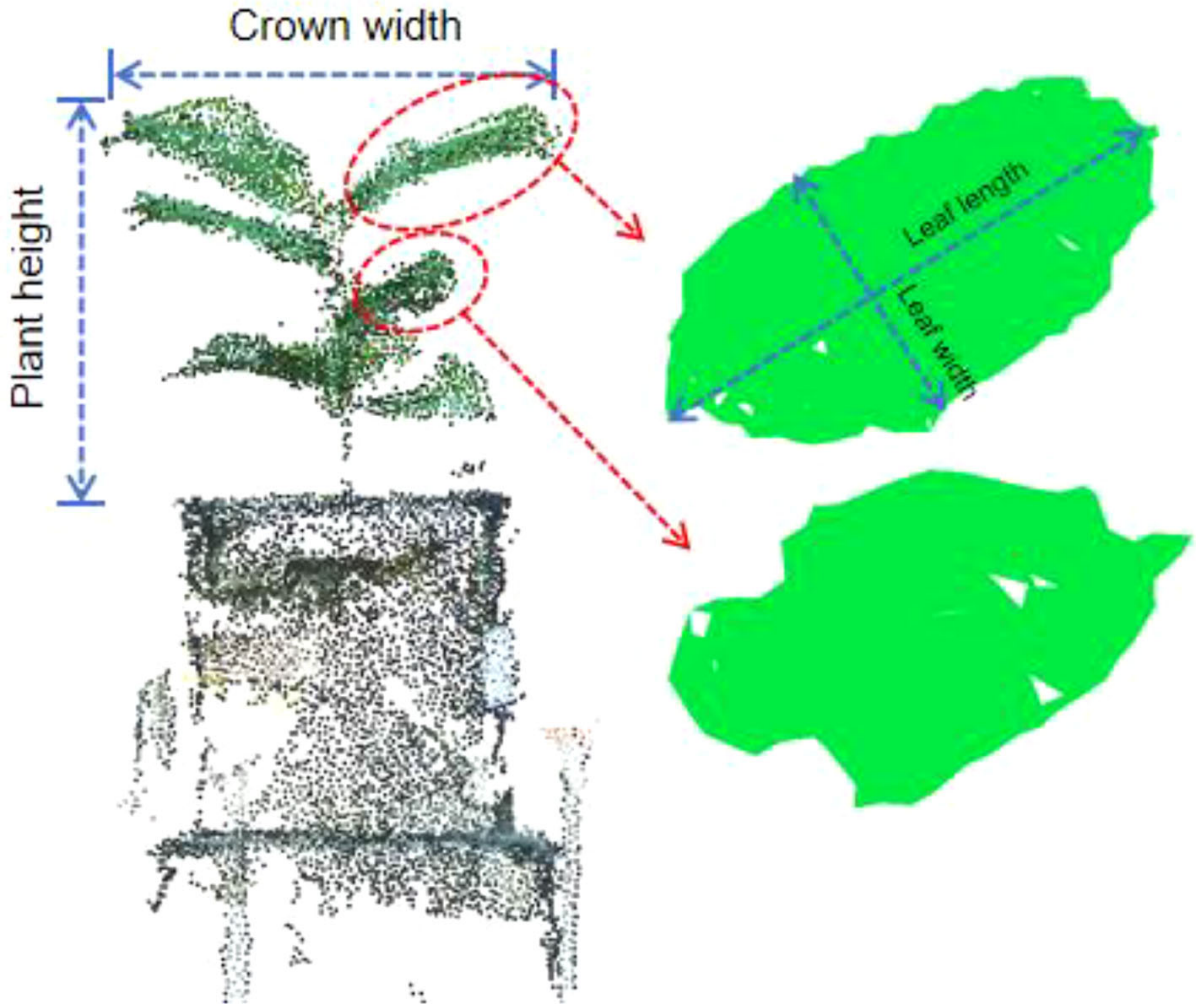
Phenotypic parameters extracted in this paper.

This study assessed phenotypic parameters at both the individual plant and leaf scales. The extracted phenotypic parameters include plant height, crown width, leaf length, and leaf width, as illustrated in **Figure 10**. When extracting leaf phenotypic parameters (leaf length and leaf width), one leaf was chosen from the upper and one from the lower part of the plant, aiming to assess the effectiveness of the method proposed.

**Figure 11:**
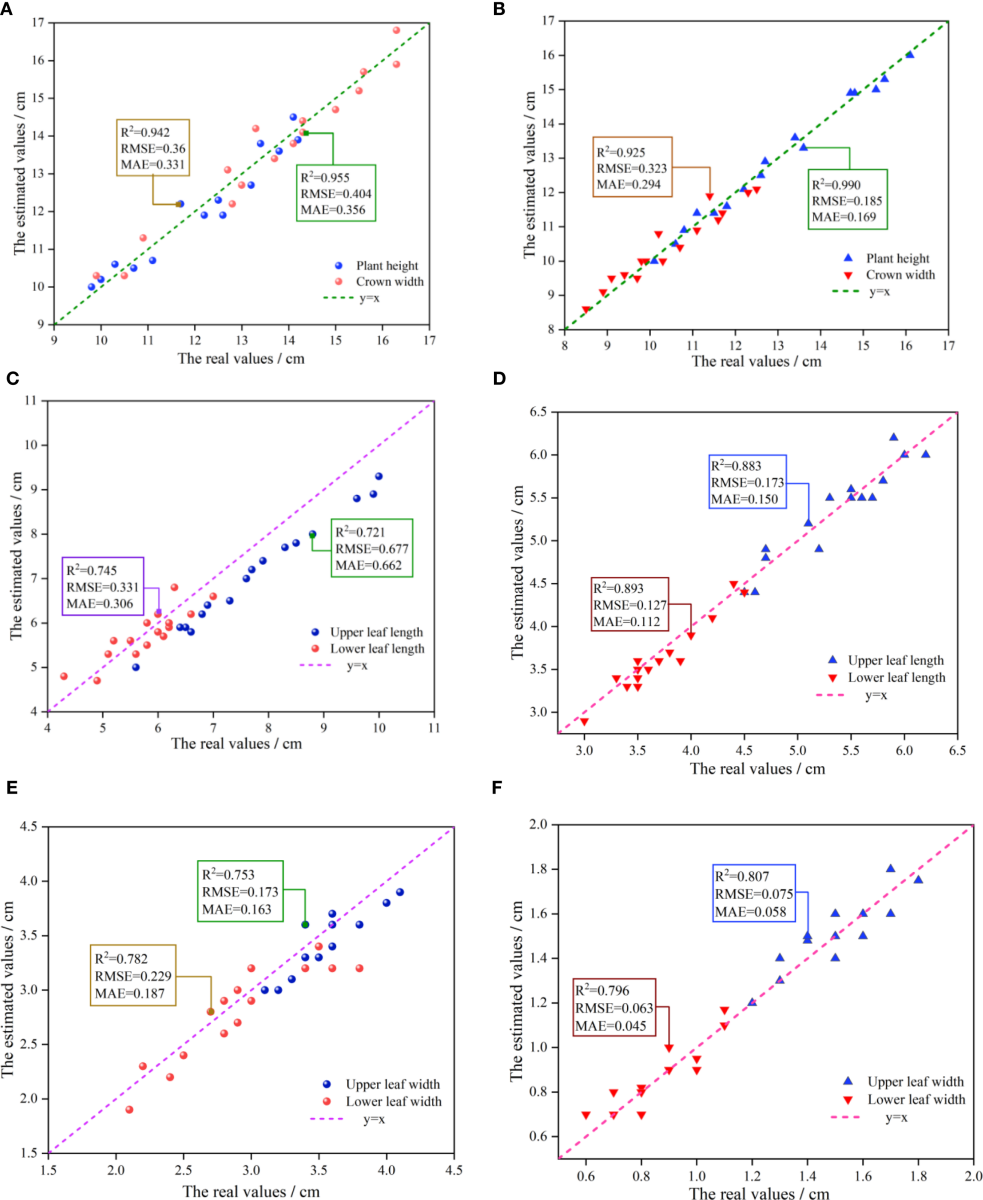
Comparative analysis of measured versus estimated values of height, leaf length, and leaf width of two *Ilex varieties*. **(A)** Scatter plot of measured versus estimated values for plant height and crown width of Ilex verticillata. **(B)** Scatter plot of measured versus estimated values for plant height and crown width of Ilex salicina. **(C)** Scatter plot of measured versus estimated values for upper and lower leaf length of Ilex verticillata. **(D)** Scatter plot of measured versus estimated values for upper and lower leaf length of Ilex salicina. **(E)** Scatter plot of measured versus estimated values for upper and lower leaf width of Ilex verticillata. **(F)** Scatter plot of measured versus estimated values for upper and lower leaf width of Ilex salicina.

The experiment was conducted on two varieties of Ilex: Ilex verticillata and Ilex salicina, with 16 samples taken from each variety. The coefficient of determination (R^2^) and root mean square error (RMSE) were employed to assess the fitting accuracy between the measured and estimated values. **Figure 11** shows the relationship between estimated and measured (real) values of phenotypic parameters for both varieties. The results indicate that: both varieties exhibit high fitting accuracy in terms of plant height and crown width, with R^2^ exceeding 0.9, and a correspondingly low RMSE of less than 0.40 cm, signifying a strong model performance. For leaf morphology (leaf length and leaf width), the R^2^ values for the upper and lower leaves of Ilex salicina are 0.883 and 0.893 respectively, with an average RMSE of approximately 0.15 cm. This significantly surpassing the 0.721 and 0.745 R² values for Ilex verticillata, which had a much higher RMSE of around 0.50 cm, indicating greater prediction error. This significant disparity is attributed to the distinct leaf shapes of the two varieties, while ilex verticillata leaves often have a curved appearance, ilex salicina leaves are generally flatter. Consequently, relying on the linear distance between two points for estimating leaf length and width for ilex verticillata can lead to significant error, as reflected in its higher RMSE. In the leaf phenotyping of ilex verticillata, the leaf length and leaf width R^2^ of the lower leaves were higher than those of the upper leaves because the lower leaves appeared to be relatively flat compared to the upper leaves, and thus the fitting accuracy was slightly higher than that of the upper leaves. For ilex salicina, the fitting accuracies of the upper and lower leaves were comparable, and the overall performance was consistent.

In terms of the morphological distribution of upper and lower leaves, it was observed that the statistical values for the upper leaves—both in terms of leaf length and width—tend to be located in the upper-right corner of the coordinate system. In contrast, the lower leaves show values in the lower-left corner, indicating that the upper leaves generally have a superior growth state. This is likely due to their more favorable exposure to light, allowing for better photosynthesis and consequently larger leaf sizes.

In summary, through the point cloud collection and registration algorithm proposed in this article, high phenotypic estimation accuracy can be achieved at individual scales (such as plant height and crown width); at the organ scale, for traits like leaf length and width. The lowest R^2^ of the estimation ranged from 0.721 to 0.99. The overall estimation accuracy for morphological phenotype parameters is satisfactory.

While the proposed workflow is effective, its applicability is limited by plant structure, scale, and environmental conditions. For plants with extremely dense and complex canopies, the six viewpoints may not fully resolve severe self-occlusion, potentially leaving significant gaps in the final model. This issue is compounded by lighting conditions; lower leaves shaded by the upper canopy can result in dark, feature-poor areas during imaging, which in turn create voids in the reconstructed point cloud and degrade accuracy. Furthermore, the registration strategy, which relies on manually placed calibration spheres, is impractical for large-scale plants like mature shrubs or trees. The method is also highly sensitive to wind, as any movement of the plant during image acquisition can cause motion blur and feature mismatching, often leading to complete reconstruction failure. Consequently, the method is optimally suited for single, small- to medium-sized plants in controlled environments.

## 5 Conclusion

This study developed and validated an integrated workflow for three-dimensional reconstruction and phenotyping of plants using stereoscopic imaging technology. First, we employed a stereo photogrammetry pipeline (SfM-MVS) to process image data captured by a stereo camera, effectively addressing the distortion issues inherent in the camera’s native depth SDK. Building on this foundation, we proposed a robust multi-view registration strategy that combines spherical markers with the iterative closest point (ICP) algorithm (SR-ICP), enabling high-precision reconstruction of complete plant point cloud models. To systematically evaluate the performance of the method, we conducted comprehensive comparative experiments. First, in terms of geometric accuracy, compared with standard MVS reconstruction, our method’s point cloud performed well in terms of average distance error (0.07 cm and 0.12 cm) and distance standard deviation (0.10 cm and 0.11 cm). Second, in terms of phenotypic parameter extraction, the results showed high correlation with manual measurements, with extremely high correlation for plant-level traits (R² > 0.92) and R² > 0.72 for organ-level traits.

Two Ilex species were selected as test subjects, with 16 plants per variety. A total of 192 measurements were conducted across four phenotypic parameters at both the individual and organ levels, providing sufficient empirical support for the method’s accuracy and reliability. While the use of spherical markers introduces some manual setup, this approach offers notable robustness in handling the complex morphology and occlusion commonly observed in plant structures. That said, the limited sample size remains a constraint, and future work should aim to expand the dataset to improve generalizability. In summary, this study presents a rigorously validated and user-friendly framework for high-fidelity 3D plant data acquisition, offering reliable technical support for plant phenotyping research and demonstrating broad application potential.

## Data Availability

The raw data supporting the conclusions of this article will be made available by the authors, without undue reservation.
